# Surface modification of doxorubicin-loaded nanoparticles based on polydopamine with pH-sensitive property for tumor targeting therapy

**DOI:** 10.1080/10717544.2018.1440447

**Published:** 2018-02-19

**Authors:** Dongdong Bi, Lei Zhao, Runqi Yu, Haowen Li, Yifei Guo, Xiangtao Wang, Meihua Han

**Affiliations:** a Institute of Medicinal Plant Development, Chinese Academy of Medical Sciences, Peking Union Medical College, Beijing, PR China;; b Life Science and Environmental Science Center, Harbin University of Commerce, Harbin, PR China;; c School of Pharmacy, Heilongjiang University of Chinese Medicine, Harbin, PR China

**Keywords:** Doxorubicin, polydopamine, folate, arginine-glycine-aspartate, pH-sensitive, tumor targeting

## Abstract

One major challenge of current surface modification of nanoparticles is the demand for chemical reactive polymeric layers, such modification is always complicated, inefficient, and may lead the polymer lose the ability to encapsulate drug. To overcome this limitation, we adopted a pH-sensitive platform using polydopamine (PDA) as a way of functionalizing nanoparticles (NPs) surfaces. All this method needed to be just a brief incubation in weak alkaline solution of dopamine, which was simple and applicable to a variety of polymer carriers regardless of their chemical reactivity. We successfully conjugated the doxorubicin (DOX)-PDA-poly (lactic-co-glycolic acid) (PLGA) NPs with two typical surface modifiers: folate (FA) and a peptide (Arg-Gly-Asp, RGD). The DOX-PDA-FA-NPs and DOX-PDA-RGD-NPs (targeting nanoparticles) were characterized by particle size, zeta potential, and surface morphology. They were quite stable in various physiological solutions and exhibited pH-sensitive property in drug release. Compared to DOX-NPs, the targeting nanoparticles possessed an excellent targeting ability against HeLa cells. In addition, the *in vivo* study demonstrated that targeting nanoparticles achieved a tumor inhibition rate over 70%, meanwhile prominently decreased the side effects of DOX and improve drug distribution in tumors. Our studies indicated that the DOX-PLGA-NPs modified with PDA and various functional ligands are promising nanocarriers for targeting tumor therapy.

## Introduction

Cervical cancer is the most common cancer among females, especially in developing countries (Dasari et al., 2015). The incidence of cervical cancer has been rising rapidly worldwide, with an estimated 275,000 deaths and 530,000 new cases every year (Goodman, 2015). In clinical practices, doxorubicin (DOX), also known as adriamycin (ADM) is a widely used treatment strategy for solid tumors, particularly cervical cancer (Zhong et al., 2017a). DOX takes effect by affecting the activity of topoisomerase II and inhibiting the synthesis of DNA to suppress cell growth (both normal and cancer cells) (Czeczuga-Semeniuk et al., 2004; Pranatharthiharan et al., 2016). Therefore, the serious side effects of DOX, such as myelotoxicity and cumulative cardiotoxicity, limited its therapeutic indexes (Singal et al., 1987; Crider et al., 2012). In order to solve this problem, many efforts have been devoted to enhancing the delivery of DOX to tumors (Lammers et al., 2008; Loomis et al., 2011; Mallick & Choi, 2014).

Recently, nanoparticle drug delivery system (NDDS) has attracted great attention and developed rapidly (Wohlfart et al., 2011). Such drug delivery system was determined to achieve greater partial drug concentration, reduce systemic toxicity and sustain drug release. Nanoparticles can reach tumors through passive targeting due to nano-size and enhanced permeability and retention (EPR) effect (Liu et al., 2016; Shaikh et al., 2016). In addition, NDDS can actively target to tumors by binding functional ligand at the nanoparticles surface to reduce the side effects on normal organs (Xing et al., 2017). To do so, sufficient target interaction is an essential requirement. Several DOX-nanoparticles have been explored and designed to improve therapeutic outcomes in malignant cancers (Zhang et al., 2015; Zavareh et al., 2016; Wang et al., 2017). Due to the water solubility of DOX, most of DOX-nanoparticles adopted the formation of liposome or PLGA nanoparticles (Yahuafai et al., 2014). However, a lot of material surfaces (e.g. PLGA), cannot be modified directly for lack of reactive functional groups, which made the surface modification of nanoparticle be quite cumbersome (Xiong et al., 2015). Moreover, if the chemical properties of polymers were changed by adding ligands, prefunctionalized polymers could lose the capability to retain and encapsulate drug (Zhong et al., 2017b). Therefore, exploring new strategies to overcome these issues is a formidable and essential task.

To overcome the complicated purification processes, to remove excess reactants and catalysts, we employed a novel, versatile and uncomplicated modification technology based on dopamine polymerization. Dopamine is a kind of traditional neurotransmitter with catechol, which can be oxidized to quinone (Li et al., 2017b). These quinones react with other catechols and/or quinones to immobilize at the solid surface, therefore, form a thin layer of polydopamine (PDA) in weak alkaline conditions (pH 8–8.5). The principle was inspired by the chemical constituents of adhesive proteins in mussels, which published in *science* (Haeshin et al., 2007). From then on, dopamine self-polymerization has been employed to introduce reactive groups on the surface of NPs (Batul et al., 2017). Park et al. also indicated that the dopamine polymerization method was a versatile and simple surface modification pathway, suitable for a variety of NP drug carriers regardless of their chemical reactivity and the types of ligands (Park et al., 2014). The reactive quinones of PDA played the role of anchoring points for further chemical macromolecules (via Michael addition and/or Schiff base reactions). The only prerequisite of this process was that nucleophilic functional groups must be possessed by the ligand molecules, such as amine and thiol (Tao et al., 2016).

Arginine-glycine-aspartate (RGD) and folate (FA) with amine as functional group are well-known targeted therapeutic ligand (Han et al., 2016; Fang et al., 2017). RGD is a cell-affinitive peptide with a three-amino acids sequence, frequently been used for targeted therapy to various tumors, such as cervical cancer, prostate cancer, breast cancer, and melanoma (Garanger et al., 2007). It could enhance the accumulation of drug-loaded RGD-nanoparticles by targeting at the αvβ3/αvβ5 integrin overexpressed in the tumor neovasculature (Wang et al., 2014). RGD-nanoparticles may affect tumor directly by extravasation and tumor cell internalization from the leaky tumor microvasculature, it also destroys tumor vasculature and subsequently isolated tumor cells from nutrient and oxygen supply (Kluza et al., 2012; Amin et al., 2015). FA, a low-molecular-weight vitamin, plays a vital role in cell survival and binds with high affinity to the folate receptor, a glycol polypeptide overexpressed in cervical cancer cells (Yang et al., 2007; Fan et al., 2012). Circulating FA-nanoparticles could bind to the cancer cells preferentially cause for normal cells possessed less folate receptor (Crider et al., 2012; Zhang et al., 2012; Haller et al., 2015).

In this study, we have modified the surface of DOX-PLGA-NP with two ligands (RGD and FA) separately for the selective binding of cancer cells. No report about the use of PDA-coated on PLGA-NP surface for targeting DOX in cervical cancer treatment is available. The novel and advanced functionalized NPs were characterized, including particle size, surface morphology, drug loading content (LC), stability, drug release profiles, and bio-distribution. The antitumor effects were investigated both *in vitro* and *in vivo*. The targeting DOX-NPs have two evident features: (1) pH-sensitive release, permitting selective intracellular drug release, and minimizing of the side effects of DOX. (2) High tumor inhibition rate (TIR) with more DOX distribution in tumor site, indicating outstanding targeted ability of NPs. We confirmed that the DOX-PLGA-NPs modified with PDA and functional ligands are promising nanocarriers for targeting tumor therapy.

## Materials and methods

### Materials

DOX was purchased from Meilun Biotech company, Dalian, China. Poly (lactic-co-glycolic acid) (PLGA, lactide/glycolide = 50:50) was obtained from Biomatrik Co. Ltd., Zhejiang, China. Bovine serum albumin (BSA, fraction V, purity 96–99%, 65,000 Da) was supplied by Sigma (Steinheim, Germany). Folate (FA, 20120417) was acquired from Sinopharm Chemical Reagent, Beijing, China. RGD peptide (CRGDRGDPDC, 96.92%, GL Biochem, Shanghai, China) was used without any disposing after received. The 3-(4,5-dimethylthiazol-2-yl)- 2,5-diphenylte-trazolium bromide (MTT) was provided by Sigma-Aldrich Co. (St Louis, MO). DiR was purchased from AAT Bioquest Inc. (Sunnyvale, CA). RPMI 1640 was developed by Roswell Park Memorial Institute (Buffalo, NY); penicillin–streptomycin and fetal bovine serum were provided by Thermo Fisher Scientific (Waltham, MA).

### Animals and cell lines

Female Balb/c nude mice (6–8 weeks old, 20 ± 2 g) were purchased from Vital River Laboratory Animal Technology Co., Ltd, Beijing, China. All the animal experiments were obtained in accordance with the regulations for animal experiments and guidelines for ethical as defined by the Institute of Medicinal Plant Development (Beijing, China). The animals were acclimated at 25 °C with standard diet ad libitum for 1 week prior to the experiments. The HeLa (cervix carcinoma) cell lines were provided by China Infrastructure of Cell Line Resource (Beijing, China) and were grown in RPMI 1640 medium (Thermo Fisher Scientific, Waltham, MA) with 10% fetal calf serum (Thermo Fisher Scientific, Waltham, MA), penicillin (100 U/mL), and streptomycin (100 U/mL) at 37 °C in 5% CO_2_ (Sanyo, Osaka, Japan).

## Preparation of nanoparticles

### Preparation of doxorubicin-loaded nanoparticles

20 mg of DOX was dissolved in 2 mL Milli-Q water as an internal aqueous phase and 200 mg of PLGA added in 3 mL of dichloromethane as the oil phase. The solution was sonicated for 70 s at 86 W to generate a stable single emulsion. Subsequently, the pre-emulsion was poured into 25 mL of 1% aqueous solution of BSA in phosphate buffer saline (PBS, pH 7.4), and the mixture was passed through a high-pressure homogenizer (JN3000, JNBIO, Guangzhou, China) at 500 bar to form the second emulsion. Thereafter, the organic solvent was removed using a rotary evaporation, and the non-capsulated excess DOX was removed by centrifugation (10 min, 13,000 rpm). The obtained nanoparticle, DOX-loaded nanoparticles was freeze-dried after the addition of 5% mannitol. The NPs can be easily labeled by putting fluorescent dye like DiR into the organic phase before emulsion.

### Preparation of doxorubicin-loaded nanoparticles coating with PDA

The DOX-loaded and PDA-coated NPs (DOX-loaded-PDA-NPs) were obtained by immersing 6 mg DOX-loaded nanoparticles (prepared at 3.3.1) in 6 mL of Tris buffer (10 mM, pH 8.5) containing 3 mg of dopamine under constant stirring at room temperature. After dopamine was stirred for 3 h, the solution turned dark, demonstrated that dopamine was successfully polymerized. Subsequently, DOX-loaded-PDA-NPs were collected by centrifugation (13,000 rpm, 10 min) for next conjugation.

### Conjugation of FA and RGD to PDA-coated NPs

RGD and FA were used, respectively, as the functional targeted ligand to conjugate to the PDA coating layers *via* the Michael addition reaction. Briefly, DOX-loaded-PDA-NPs (prepared at 3.3.2) was resuspended in Tris buffer (10 mM, pH 8.5), which contains different 2 mg/mL ligands (RGD or FA). After 0.5 h of incubation under constant stirring at room temperature, the functionalized NPs were centrifuged and washed with deionized water for three times, then freeze-dried after addition of 5% mannitol. The functionalized NPs were designated as DOX-PDA-FA-NPs and DOX-PDA-RGD-NPs according to the ligand used for the functionalization.

## Characterization of functionalized NPs

### Particle size, zeta potential, and morphology

DOX-NPs, DOX-PDA-NPs, DOX-PDA-FA-NPs, and DOX-PDA-RGD-NPs were suspended in phosphate buffer (pH = 7.4), and their sizes, zeta potentials, and polydispersity index (PDI) were analyzed in triplicate (12 scans) by Dynamic Light Scattering (DLS, Zeta sizer Nano ZS, Malvern Instruments, Worcestershire, UK) at room temperature. The surface morphology of NPs was observed by a JEM-1400 electron microscope (TEM, JEOL Ltd., Tokyo, Japan). One drop of the nanoparticles was placed on a 300-mesh copper grid, then air-dried and stained with 2% (w/v) uranyl acetate for observation under electron microscope.

### Stability of DOX-NPs in various physiological solutions

DOX-PDA-FA-NPs and DOX-PDA-RGD-NPs (2 mg/mL) were mixed (1:1, v/v) with 1.8% NaCl and 10% glucose, respectively, to obtain an isotonic solution and then were incubated at 37 °C. At specific time intervals, 1 mL sample was removed and analyzed for size changes and particle distribution. Each experiment was performed in triplicate.

### Stability of DOX-NPs in rat plasma

An *in vitro* plasma stability test was implemented to explore whether plasma components (enzymes and serum albumin) could have an interaction with DOX-NPs and induce aggregation. DOX-PDA-FA-NPs and DOX-PDA-RGD-NPs (0.5 and 2 mg/mL) were mixed with rat plasma (1:4, v/v) and incubated at 37 °C. At specific time intervals, 1 mL of the sample was removed and analyzed (size change and particle distribution). Each experiment was performed in triplicate.

### Fourier transform infrared spectroscopy analysis

The Fourier transform infrared (FT-IR) spectroscopy analysis of drug-free NPs, drug-free PDA-NPs, drug-free PDA-RGD-NPs, and drug-free PDA-FA-NPs was recorded by FT-IR spectrophotometer (Thermo Nicolet, Madison, WI) using KBr.

### X-ray photoelectron spectroscopy

The X-ray photoelectron spectroscopy (XPS) of the drug-free NPs, drug-free PDA-NPs, drug-free PDA-RGD-NPs, and drug-free PDA-FA-NPs was obtained by a Kratos Axis Ultra DLD spectrometer (Kratos Analytical Ltd, Manchester, UK) with monochromatic Al Kα radiation (*hv* = 1486.58 eV). Survey and high-resolution spectra were collected at a fixed analyzer pass energy of 160 and 20 eV, respectively. Binding energy values were referenced to the Fermi edge and charge correction was performed setting the C 1s peak at 284.80 eV.

### Drug loading capacity

5 mg freeze-dried DOX-NPs, DOX-PDA-NPs, DOX-PDA-FA-NPs, and DOX-PDA-RGD-NPs were dissolved in 1 mL of methanol under vigorous vortexing. The supernatants were transferred to 5 mL of mobile phase consisting of methanol and 0.2% phosphoric acid (50:50, v/v). The loading capacity (LC) of the modified NPs was determined by high-performance liquid chromatography (HPLC; Ultimate 3000, DIONEX, Germering, Sunnyvale, CA, USA). A reverse-phase C18 column (5 μm, 250 × 4.6 mm^2^, Waters Symmetry, Milford, MA) was used at 25 °C. The flow rate of mobile phase was set at 1.0 mL/min and the injection volume was 20 uL. The detection was performed by UV–Vis absorption at 266 nm. Measurements were carried out three times for each batch. The LC of modified NPs are calculated by the following Equation (1).

(1) $$LC\;\left(\%\right)=\frac{Weight\;of\;DOX\;in\;NPs}{\;Weight\;of\;NPs}\times 100\%$$

## *In vitro* drug release

The dialysis bag diffusion method was used to analyze the *in vitro* drug release of the DOX-NPs, DOX-PDA-NPs, DOX-PDA-FA-NPs, and DOX-PDA-RGD-NPs (2 mL, 1 mg/mL), which was investigated in phosphate-buffered saline with different pH. Briefly, NPs were putted into a regenerated cellulose dialysis bag (MWCO, 8000–14000, Sigma, St. Louis, MO). The closed bag was then put into a centrifuge tube and immersed into 50 mL of release medium PBS pH = 7.4, 6.5, 5.0, 0.1 mol/L). The tube was transferred into an orbital water bath and shaken at 120 rpm at 37 °C. At certain time points, 1 mL of release medium was removed for HPLC analysis and replaced with fresh PBS solution. Each batch of experiments was performed in triplicate. The accumulative releases of drug from DOX-NPs, DOX-PDA-NPs, DOX-PDA-FA-NPs, and DOX-PDA-RGD-NPs were plotted against time.

## *In vitro* cytotoxicity assay

The cytotoxicity of the DOX-loaded NPs was evaluated by the MTT assay. Typically, HeLa cells in the logarithm growth period were seeded on 96-well plates (8000 cells/well) and incubated overnight in a humidified atmosphere of 5% CO_2_ at 37 °C. Various concentrations of 200 μL DOX-loaded NPs (DOX-NPs, DOX-PDA-NPs, DOX-PDA-FA-NPs, and DOX-PDA-RGD-NPs), competitive inhibition groups (the physical mixtures of FA and DOX-PDA-FA-NPs, physical mixtures of RGD and DOX-PDA-RGD-NPs) or free DOX solution (dissolved in 0.9% NaCl) were added and incubated for 24 and 48 h. The cytotoxicity of the blank NPs was also determined. Then 20 μL of MTT solution (5 mg/mL in PBS) was added to the cells and the plate was incubated for 4 h. The medium was removed and 150 μL of DMSO was mixed to each well and the maximum absorbance was detected at 570 nm using an ELISA plate reader (Biotek, Winooski, VT). The cell inhibitory rate is calculated according to the following Equation (2):

(2) $$Cell\;inhibitory\;rate\;(\%)=1-OD_{e}/OD_{c}\times 100\%$$

where OD_e_ is the mean optical density of the experimental group and OD_c_ is the mean optical density of the control group.

The half maximal inhibitory concentration (IC_50_) was calculated using the GraphPad Prism software version 5 (GraphPad Software, Inc., La Jolla, CA) by the sigmoidal dose-response variable curve-fitting method.

## *In vivo* biodistribution study

Female Balb/c nude mice at 6 weeks of age were injected subcutaneously in the right armpit with 0.2 mL of HeLa cells (5.0 × 10^6^ cells/mL). The HeLa tumor-bearing mice were randomly divided into four groups (five mice per group) and were intravenously injected with DOX/DiR-NPs, DOX/DiR-PDA-NPs, DOX/DiR-PDA-FA-NPs, and DOX/DiR-PDA-RGD-NPs at 2 mg/kg (Dox-eq. dose, DOX: Dir = 40:1), respectively. During the experiments, the whole-body fluorescence images were recorded at 0.5, 4, 8, 12, and 24 h using the IVIS Living Image^®^ version 4.4 (Caliper Life Sciences, Hopkinton, MA). At the end of the experiments, their organs (heart, liver, spleen, lung, kidney, and tumor) followed by washing with 0.9% NaCl for the *ex vivo* imaging. Living Image software version 4.2 (Caliper Life Sciences, Hopkinton, MA) was used in quantitative analysis. All the mice were imaged with identical instrument settings, and all the groups at each time point were under the same scale bar.

## *In vivo* antitumor activity in HeLa tumor-bearing mice

*In vivo* anticancer effect of DOX-NPs was performed on HeLa tumor-bearing Balb/c nude mice. Briefly, the mice were inoculated subcutaneously with 0.2 mL of HeLa cells suspended in a culture medium at a density of 5.0 × 10^6^ cells/mL in the right armpit under sterile conditions. When the volume of tumor reached 100 mm^3^, the HeLa tumor-bearing mice were randomly divided into six groups, and each group comprised eight mice. The mice were injected intravenously every two days (Liu et al., 2012; Wang et al., 2016) with normal saline (as a negative control), DOX (as a positive control), DOX-NPs, DOX-PDA-NPs, DOX-PDA-FA-NPs, and DOX-PDA-RGD-NPs, at equivalent DOX concentrations of 4 mg/kg, respectively. During the entire administration process, the volume of tumors and the body weight were measured every two days. The mice were given a euthanasia and dissected on the 12th day of treatment. The tumors were harvested and weighed. Tumor volume is calculated using Equation (3):

(3) $$V=\frac{a\times b^{2}}{2}\;$$

where ‘*V*’ is the tumor volume, ‘*a*’ is the length of the major axis, and ‘*b*’ is the length of the minor axis.

The TIR is calculated using the following Equation (4):

(4) $$TIR\;\left(\%\right)=\frac{1-W_{e}}{W_{n}}\times 100\%$$

where ‘*W*_e_’ is the mean tumor weight of the experimental group, and ‘*W*_n_’ is the mean tumor weight of the negative control group.

## Statistical analysis

The statistical analysis of the experimental groups was performed using the independent-samples t-test with the software of IBM SPSS Statistics version 19 (IBM Co., Armonk, NY). *p < .05 or less was considered statistically significant.

## Results and discussion

### Preparation of nanoparticles

**Figure 1.**
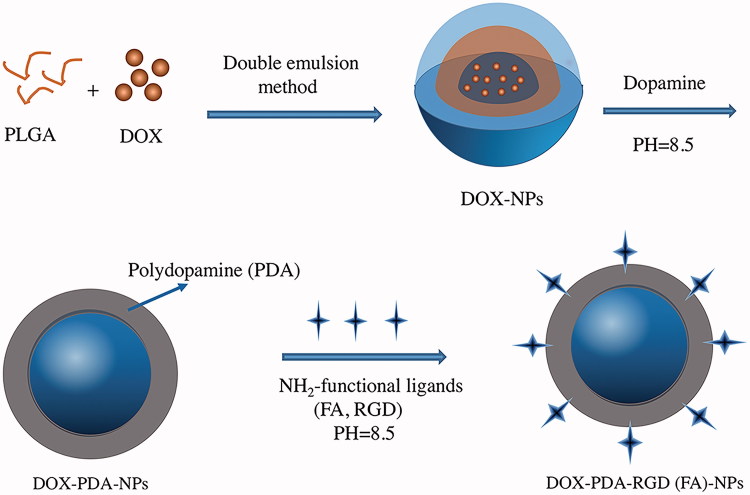
Schematic representation of the preparation procedure for targeted and DOX-PDA-FA (RGD)-NPs.

DOX has relatively good solubility in water and poor solubility in organic solvents, whence we prepared the nanoparticles using double emulsions (W-O-W) method. The preparation of DOX-PDA-FA-NPs and DOX-PDA-RGD-NPs are exhibited in Figure 1. PLGA was dissolved in dichloromethane (organic phase) and emulsified with aqueous solution of DOX to form a water in oil emulsion. Of 1% solution of BSA was used instead of PVA in this study as a stabilizer (Wohlfart et al., 2011).

The DOX-NPs were then immersed in the dopamine solution to form a tight PDA surface layer. The weak alkaline condition is an essential process for oxidation of dopamine and conjugation of ligand (FA and RGD) to PDA coated NPs according to Michael addition or Schiff-base reaction (Xing et al., 2017). While dopamine was added, the suspensions gradually turned dark suggesting that dopamine was successfully polymerized (Hong et al., 2015).

### Characterization of functionalized NPs

**Figure 2.**
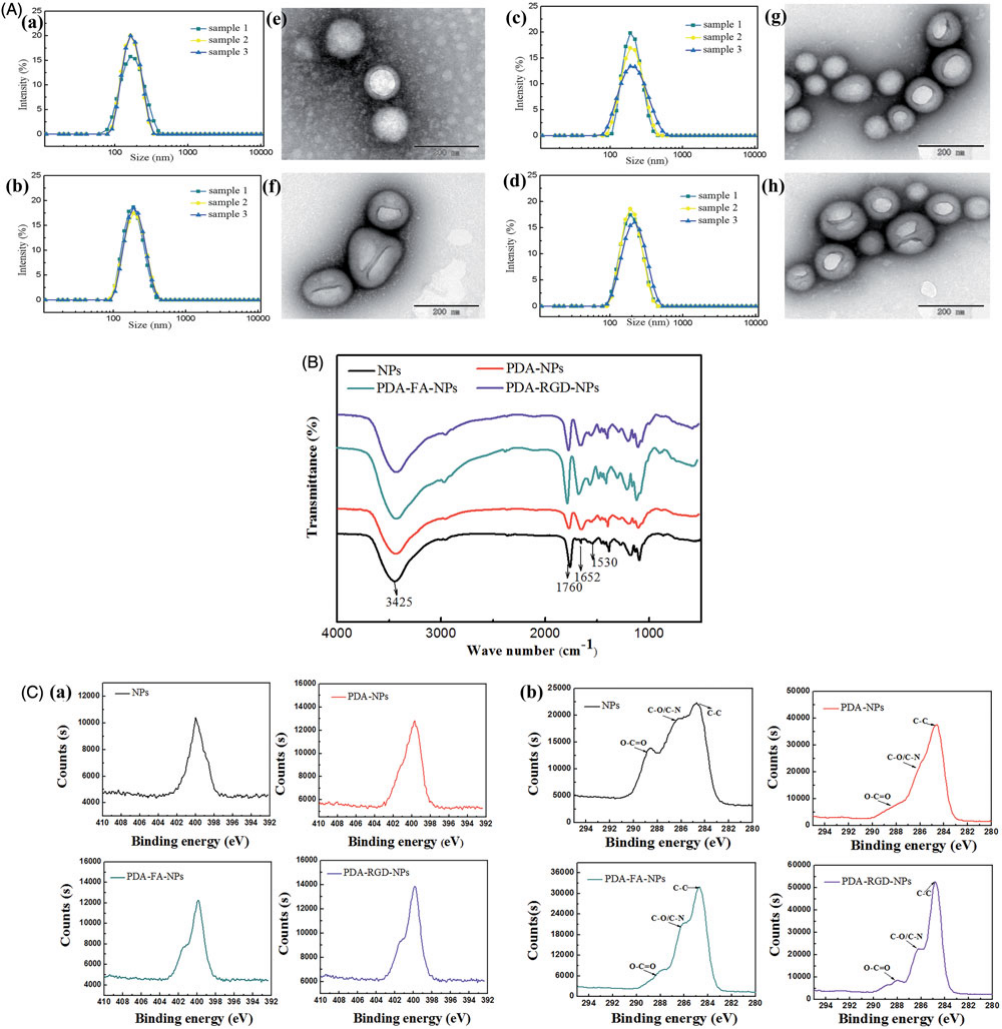
TEM images, DLS size distribution, Fourier transform infrared spectra and XPS spectra analysis of DOX-NPs, DOX-PDA-NPs, DOX-PDA-FA-NPs, and DOX-PDA-RGD-NPs (*n* = 3). A(a–d): TEM images. (e–h): DLS size distribution. B: Fourier transform infrared spectra. C(a): XPS: narrow scan for N1s peaks. (b): XPS: narrow scan for C1s peaks.

The size and surface properties of NPs have indispensable position in drug release, *in vivo* pharmacokinetics and cellular uptake (Xu et al., 2016). Small size nanoparticles can circulate through the microvascular bed of a tumor and extravasate into the perivascular space by convective transport through the endothelium and retained at the site, which was known as the EPR effect (Loomis et al., 2011). As shown in Table S1, the obtained DOX-NPs had a mean particle size of 162.9 nm, PDI value of 0.193. A small PDI value (<0.2) indicated that the size distribution of DOX-NPs was narrow. Meanwhile, the size of DTX-PDA-NPs, DTX-PDA-RGD-NPs, and DTX-PDA-FA-NPs increased nearly 30 nm, which may be due to the thin PDA films (Zavareh et al., 2016). The size distribution of DOX-NPs, DOX-PDA-NPs, DOX-PDA-FA-NPs, and DOX-PDA-RGD-NPs is illustrated in Figure 2(A(a–d)).

Zeta potential is crucial for the stability of the NPs, the zeta potential of functionalized NPs tended to more negative then DOX-NPs. This phenomenon may be caused by PDA coating, which is a kind of negative polyelectrolytes (Huang et al., 2015). Moreover, such situation was more obvious via FA conjugations, which indicated this continuity of modification did improve the dispersity of nanoparticles in suspension (Ai et al., 2017).

The drug LC of DOX-PDA-RGD-NPs and DOX-PDA-FA-NPs was a bit lower than DOX-NPs and DOX-PDA-NPs, indicating that drug molecules were merely lost during the preparation of targeted nanoparticles.

In this study, the morphologies and structures of DOX-loaded nanoparticles were investigated by TEM, FT-IR spectroscopy, and XPS, respectively. The TEM images of DOX-NPs, DOX-PDA-NPs, DOX-PDA-FA-NPs, and DOX-PDA-RGD-NPs is illustrated in Figure 2(A(e–h)), which revealed that nanoparticles were spherical and regular in shape. Interestingly, it could be obviously visualized that thin spherical films on the surface of DOX-PDA-NPs, DOX-PDA-FA-NPs, and DOX-PDA-RGD-NPs, which displays that PDA films were successfully deposited on the surface of DOX-NPs through an oxidative polymerization reaction (Zhang et al., 2017).

The high-resolution XPS spectra further illustrated the binding energies and the relative percentages of N1s and C1s on the nanoparticles. In Figure 2(C(a)), both PDA-FA-NPs and PDA-RGD-NPs emerged two nitrogen peaks (N 1s) at −399.56 eV (nonprotonated amine groups) and −401.8 eV (protonated amine groups) in XPS spectra. However, the bare NPs and PDA-NPs only showed one nitrogen peak at −399.7 eV, and the peak area of PDA-NPs was obviously larger than bare NPs (18004 vs. 14630 eV), which further demonstrated the presence of a PDA layer and targeting ligand (FA and RGD) on the surface of NPs after modification (Park et al., 2014; Feng et al., 2016). To Confirm the coating of PDA and the functionalization of NPs, we used narrow XPS scan for C1s peaks (Figure 2C(b)). The C 1s of bare NPs has three resolved peaks: O–C=O (288.21 eV), C–O/C–N (286.06 eV), and C–C (284.77 eV), however, after PDA modification of the NPs, a large proportion of the peak O–C=O (288.71 eV) was shielded (Kim et al., 2017; Šírová et al., 2017). For the PDA-FA-NPs and PDA-RGD-NPs, the intensity of the peak of O–C=O decreased, but the intensity of these peaks of C–O/C–N and C–C peaks increased, which further demonstrated the ligand FA and RGD were conjugated to PDA-NPs successively (Xiong et al., 2015; Zhao et al., 2017).

As proof, FTIR also provides information with higher detection sensitivity to characterize the surface chemical group composition of NPs. Figure 2(B) presents the FTIR spectra of the drug-free NPs, PDA-NPs, PDA-FA-NPs, and PDA-RGD-NPs. Both the bare nanoparticles and dopamine-coated nanoparticles exhibit a strong peak at 3425 cm^−1^, which could be assigned to the stretching vibration of the hydroxyl groups including surface adsorbed water (Cheng et al., 2017a). In addition, the 3425 cm^−1^ band of PDA-NPs, PDA-FA-NPs, and PDA-RGD-NPs were increased than NPs, which may owe to the N–H and O–H stretching modes of FA, RGD, and PDA (Zavareh et al., 2016). As for NPs, the distinct peaks at 1760 cm^−1^ was assigned to the carbonyl band of PLGA (Sheng & Tao, 2016), for PDA-NPs, the 1760 cm^−1^ band significantly decreased, which indicated that the PDA-coating was conjugated on NPs. Furthermore, the absorption band at 1760 cm^−1^ of PDA-FA-NPs and PDA-RGD-NPs were increased, consistent with the exist of carbonyl in RGD and FA, suggesting the targeting group conjugated on PDA-NPs (Li et al., 2017a). The characteristic band at 1652 cm^−1^ was attributed to the C=C bond stretching vibration of PDA. The broadband spanning 900–1300 cm^−1^ might originate from the aromatic skeletal vibrations (Fan et al., 2015). In summary, the FT-IR spectroscopy and XPS studies demonstrated the successful coated of PDA film and conjugation of FA and RGD on the surface of NPs.

### Stability of DOX-NPs in various physiological solutions

The DOX-PDA-FA-NPs and DOX-PDA-RGD-NPs were stable in physiological saline, isotonic glucose. There were no apparent particle size enlargement and aggregation after incubation at 37 °C for 24 h (Figure S1). There were varieties of serum albumins and enzymes in plasma that can be adhered on the surface of nanoparticles and between times resulting in blood blockage or aggregation (Ji et al., 2017). DOX-PDA-FA-NPs and DOX-PDA-RGD-NPs maintained their size during 12 h of incubation with rat plasma, suggesting their good plasma stability. The result meant DOX-PDA-FA-NPs and DOX-PDA-RGD-NPs both meet the demand of intravenous injection.

### Targeting DOX-NPs possessed pH-sensitive property

**Figure 3.**
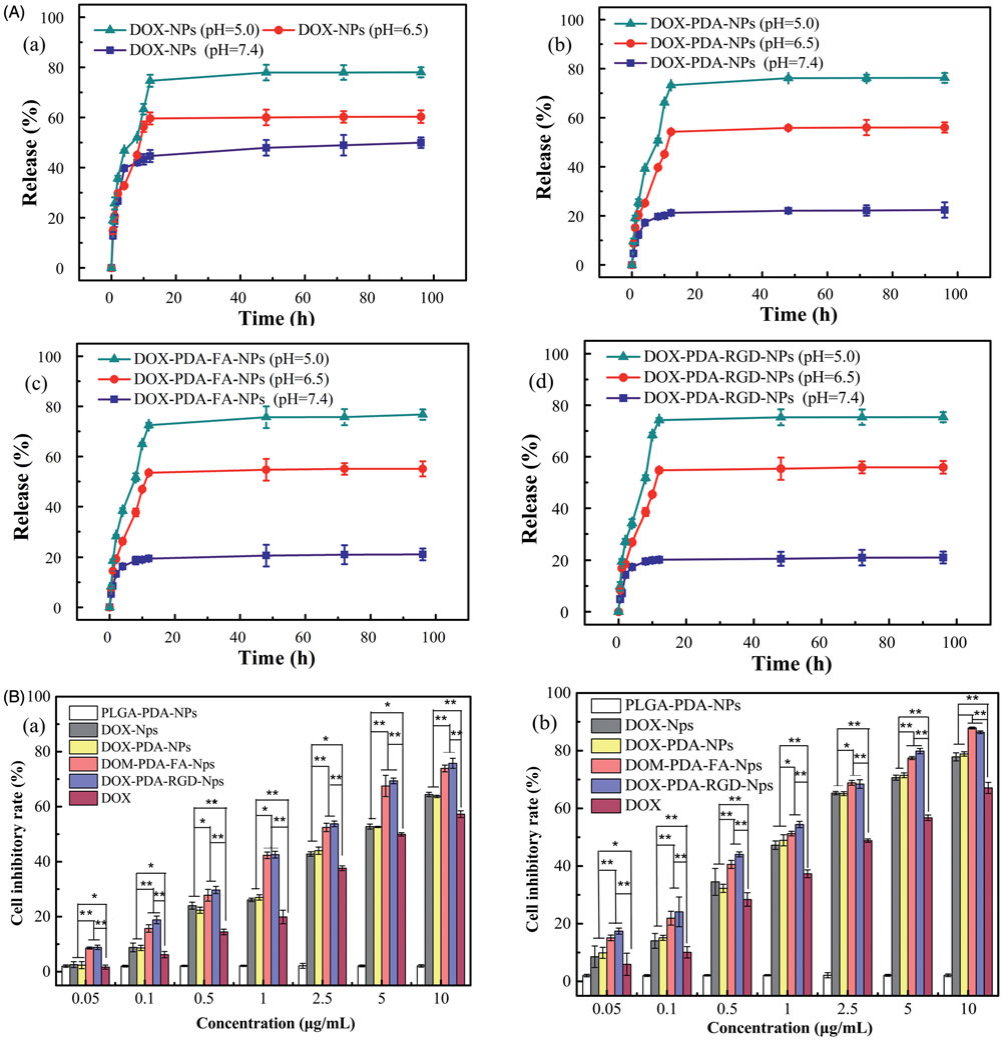
*In vitro* drug release profile of NPs in PBS with different pH value and cell inhibitory rate of HeLa cells incubated with A(a): DOX-NPs, (b): DOX-PDA-NPs, (c): DOX-PDA-FA-NPs, and (d): DOX-PDA-RGD-NPs (*n* = 3). B(a): HeLa cells inhibitory rate at 24 h and (b) HeLa cells inhibitory rate at 48 h (*n* = 6). *p < .05; **p < .01.

The functionalized particles slowly released DOX over 48 h at 37 °C under pH 7.4, 6.5, and 5.0 PBS solutions, which was both time- and pH-dependent (Figure 3(A)). As well-known, natural pH gradients in the endosomal or lysosomal of tumor cells is 5.0–6.5 and in tumor microenvironment is 6.5–7.2 (Meng et al., 2014). PH-sensitive nanoparticles could retain drug during circulation in body fluid while actively releasing it at the tumor site or in the endosomal or lysosomal of target tumor cells, which can enhance the anti-tumor effect as well as reduce potential damage to normal cells (Cheng et al., 2017b). The cumulative dissolution profiles of nanoparticles are shown in Figure 3(A). At pH 7.4, only 22.40% of drug was released from DOX-PDA-NPs (21.13 and 21.07% for DOX-PDA-FA-NPs and DOX-PDA-RGD-NPs), respectively, over the course of 48 h, whereas at pH 6.5 they showed higher release content with 56.01% (55.03 and 55.87%) and at pH 5.0 up to 76.12% (75.68 and 76.30%). DOX-NPs showed a little burst release in the initial 5 h; however, after surface modification with PDA, it evinced a decrease in initial burst release.

The result indicated that PDA-coated NPs exhibited a higher DOX release rate at endosomal pH (5.0–6.5) as compared with physiological pH (7.4). This phenomenon may attribute to PDA-coated aggregates, which were able to preserve the structures of the nanoparticles at physiological pH, but unlock the channel of the NPs in acidic conditions (Wang et al., 2016; Zong et al., 2016). Nevertheless, for DOX-NPs, we also discovered that the DOX release rate slightly increased with the decrease of pH. The result is due to DOX possess the protonation of the amino group, which could gave DOX a positive charge to enhance its solubility in acidic conditions. The acidity-induced dissolution of DOX in aqueous environments results in a faster drug release (Mu et al., 2017). The pH-sensitive DOX-NPs drug release processes enabled supervision of intracellular drug release and minimizing the side effects of DOX.

### Targeting DOX-NPs exhibited greater cytotoxicity on HeLa cells

To evaluate the cytotoxicity of various DOX formulations against HeLa cells, the MTT assay was carried out. HeLa cells were selected for the superior levels of folate receptor and αvβ_3_/αvβ_5_ integrin present in them. The cells were treated with DOX, DOX-NPs, DOX-PDA-NPs, DOX-PDA-FA-NPs, DOX-PDA-RGD-NPs, free FA with DOX-PDA-FA-NPs, free RGD with DOX-PDA-RGD-NPs at different concentrations for 24 and 48 h. The result indicated that the cytotoxicity of all DOX formulations was incubation time- and concentration-dependent (Figure 3(B)). All the nanoparticles exhibited a much stronger tumor growth inhibition than DOX at all concentrations. Furthermore, DOX-PDA-FA-NPs and DOX-PDA-RGD-NPs exhibited better *in vitro* antitumor efficacy than others, on account of targeted ligand FA and RGD well specifically recognized and killed HeLa cells signally and the ligand may have an outstanding targeting effect on the NP surface (Zhang et al., 2012; Wang et al., 2014). However, the drug-free NPs with various concentrations showed no significant cytotoxicity, which indicated excipients (PLGA and PDA) seems to be nontoxic, highly biocompatible, and safe in cell culture (Ding et al., 2017). To find out whether folate (RGD)-mediated endocytosis effectively contributes to the equipotent cytotoxicity of DOX-PDA-FA (RGD)-NPs, cell cytotoxicity assay were performed in the presence of 1 mM FA (RGD) (Wang et al., 2013). The competitive inhibition groups showed less cytotoxicity than targeting group when cells treated on the same dosage (Figure S2). Therefore, the enhanced cytotoxicity of DOX-PDA-FA (RGD)-NPs could be attributed to the fact that FA (RGD) played a key role in promoting the cellular uptake of targeting NPs into FR (αvβ_3_/αvβ_5_ receptors)-positive tumor cells (Hong et al., 2017).

These results could be quantitatively demonstrated in terms of IC_50_ values, which is defined as the drug inhibitory concentration causing 50% tumor cell mortality in a designated period, and listed in Table 1. The IC_50_ values of DOX-PDA-FA-NPs and DOX-PDA-RGD-NPs were 1.86 ± 0.17, 0.80 ± 0.06, and 1.66 ± 0.16, 0.79 ± 0.13 μg/mL after incubation 24 and 48 h, respectively, which were significantly lower than other DOX formulations, free FA competitive inhibition groups (9.033 ± 0.81) and free RGD competitive inhibition groups (5.651 ± 0.32).The DTX-NPs and the DTX-PDA-NPs also showed similar cytotoxicity at various concentrations, further suggested that the PDA coated did not influence the cell viability, being nontoxicity and biocompatible (Ding et al., 2017). Taken together, targeted DOX-PDA-FA-NPs and DOX-PDA-RGD-NPs could be used as a promising drug delivery system with their extremely high-efficiency *in vitro* therapeutic effects. It could be ascribed to the αvβ_3_/αvβ_5_ receptors and folate receptor are over-expressed on the surface of HeLa cell, which was in agreement with the previous reports (Wang et al., 2014).

**Table 1.** IC_50_ values of DOX-NPs, DOX-PDA-NPs, DOX-PDA-FA-NPs, DOX-PDA-RGD-NPs, and DOX on HeLa cells following 24 and 48 h incubation, respectively (*n* = 8).

	IC_50_ (μg/mL)				
Incubation time (h)	DOX-NPs	DOX-PDA-NPs	DOX-PDA-FA-NPs	DOX-PDA-RGD-NPs	DOX
24	4.10 ± 0.55	4.07 ± 0.79	1.86 ± 0.17	1.66 ± 0.16	5.73 ± 0.52
48	1.24 ± 0.12	1.19 ± 0.11	0.80 ± 0.06	0.79 ± 0.13	2.82 ± 0.28

### Targeting DOX-NPs exhibited higher accumulation at the tumor site

**Figure 4.**
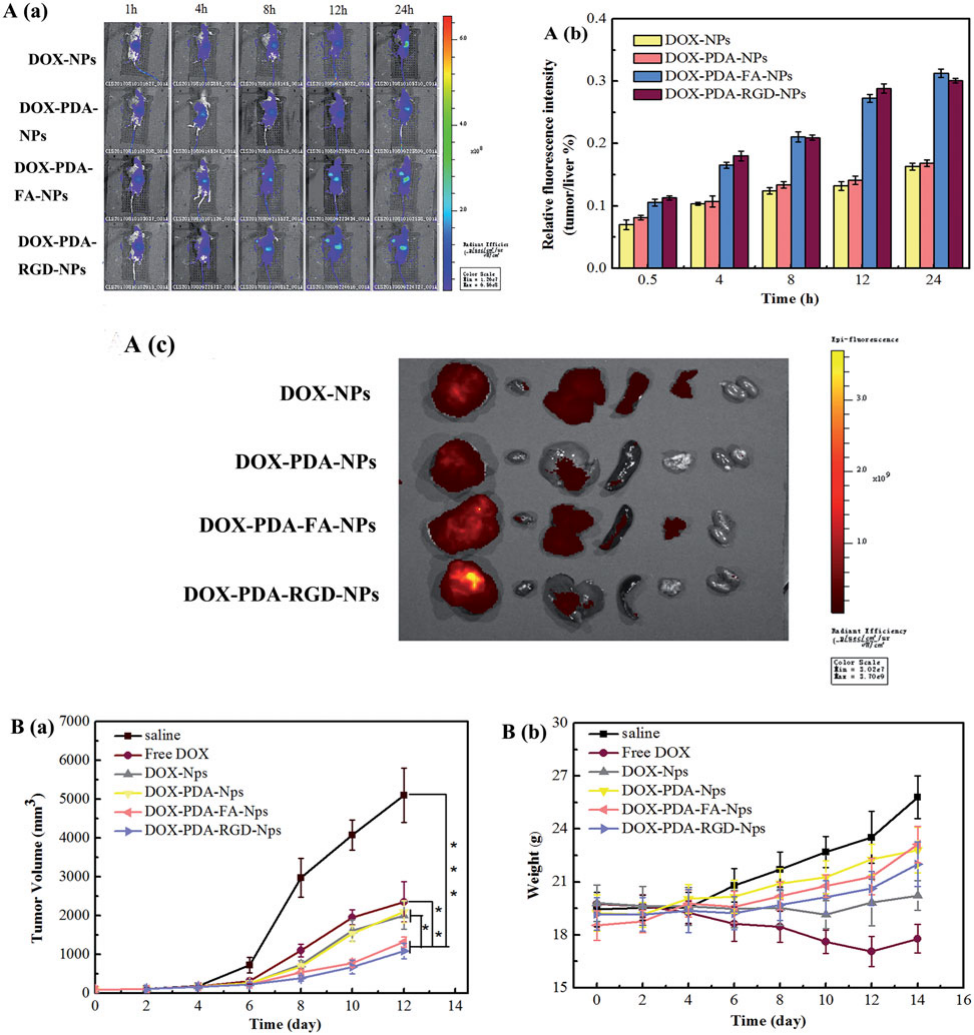
The distribution and *in vivo* antitumor activity of DOX, DOX-NPs, DOX-PDA-NPs, DOX-PDA-FA-NPs, and DOX-PDA-RGD-NPs toward HeLa tumor-bearing mice. A(a): The distribution of different DOX formulations in HeLa tumor mice at various time point. (b) Average fluorescence intensity of tumors/average fluorescence intensity of liver at various time point. (c) *In vivo* fluorescence images of major organs of HeLa tumor-bearing nude mice at the end of the experiment (from left to right: tumor, heart, liver, spleen, lung, and kidney). (All data represent the mean ± SD, *n* = 5). B(a) tumor growth curves. (b) Changes of body weight. (mean ± SD, *n* = 8). *p < .05, **p < .01, ***p < .001.

To evaluate the targeting capability and *in vivo* distribution of DOX-NPs, DOX-PDA-NPs, DOX-PDA-FA-NPs, and DOX-PDA-RGD-NPs, the drugs were intravenous (iv) injected into the Balb/c nude mice bearing a subcutaneous HeLa tumor. Figure 4(A(a)) demonstrates the *in vivo* fluorescence intensity of mice at different time points after iv administration. All DOX formulations were mainly concentrated in the liver within the first 1 h. Then, increasing fluorescence began to accumulate in the tumor and reached the maximum. The signal intensity in tumor of DOX-PDA-FA-NPs and DOX-PDA-RGD-NPs was stronger over 8 h compared with other groups. This result illustrates that the amount of DOX at tumor site in the DOX-PDA-FA-NPs and DOX-PDA-RGD-NPs groups were higher than groups without targeting (DOX-NPs and DOX-PDA-NPs), which could due to the folate receptor and αvβ3/αvβ5 integrin-mediated targeting (Amin et al., 2015; Fan et al., 2015). What’s more, the relatively high fluorescence density in the liver for all of these formulations could interpret as the uptake of NPs by macrophages of the reticuloendothelial system, particularly the Kupffer cells in the liver (Zhang et al., 2012). The average fluorescence intensity of tumors/average fluorescence intensity of liver at various time points are shown in Figure 4(A(b)), with the result that DOX-PDA-FA-NPs ≈ DOX-PDA-RGD-NPs > DOX-NPs ≈ DOX-PDA-NPs, which clearly indicated the *in vivo* targeting ability of DOX-PDA-FA-NPs and DOX-PDA-RGD-NPs.

In addition, compared with *in vivo* imaging, *in vitro* imaging could detect the fluorescence signal more accurate by reducing the fluorescence noise of skin and the fluorescence decay in the process of penetrating tissue. The fluorescence signals of major organs harvested from all groups of nude mice at 24 h post-injection is in Figure 4(A(c)), from left to right were tumor, heart, liver, spleen, lung, and kidney. The result indicated the fluorescence mainly distributed in the in the tumor and liver, secondly in heart and spleen, least in kidney, and lung. The accumulation of DOX-PDA-FA-NPs and DOX-PDA-RGD-NPs were much higher at the tumor site. All results suggested the *in vivo* targeting ability of DOX-PDA-FA-NPs and DOX-PDA-RGD-NPs. Therefore, the *in vivo* therapeutic efficacy in tumor-bearing mice was investigated subsequently.

### Higher tumor inhibition rate and lower toxicity of targeting DOX-NPs

To investigate *in vivo* the antitumor efficacy of different formulations of DOX, BALB/c nude mice were subcutaneously inoculated into the right flank with 5.0 × 10^6^ cells/mL HeLa cells. When the tumor volumes approximately were 100 mm^3^, the mice were divided into six groups (*n* = 8) and treated with DOX, DOX-NPs, DOX-PDA-NPs, DOX-PDA-FA-NPs, and DOX-PDA-RGD-NPs at 4 mg/kg DOX dose or saline through intravenous injection. As shown in Figure 4(B(a)), noteworthy tumor growth inhibition was revealed in all groups receiving DOX. The tumor volume in the saline control group increased rapidly. The entire groups of tumor treated with DOX-NPs showed a significantly smaller tumor volume than the saline control group, which possibly due to the absence of the EPR effect. The group treated with DOX-PDA-RGD-NPs (1090 mm^3^) and DOX-PDA-FA-NPs (1424 mm^3^) were similar and showed the lowest growth rate among all the groups, which was inconsistent with *in vitro* anti-cancer effect. Table 2 displays the tumor weight, tumor inhibitory rate, liver index, and spleen index against HeLa tumors for all the groups. It also showed that DOX-PDA-RGD-NPs and DOX-PDA-FA-NPs were superior to free DOX, DOX-NPs, and DOX-PDA-NPs regarding TIR (75.11, 70.82 vs. 53.59%, 59.01, 59.66%, *p* < .05).The liver and spleen index were used to evaluate whether the liver and spleen are damaged by chemotherapy (Yahuafai et al., 2014). According to the consequence (Table 2), the liver and spleen index of DOX reduced extremely compared with the saline group (*p* < .01), while other NPs nearly had no differences. This phenomenon indicated that the formulation of nanoparticles could decrease the toxicity of DOX in spleen and liver, which may due to the EPR effect and the sustained slow release of nanoparticles (Šírová et al., 2017).

**Table 2.** The *in vivo* antitumor effects of different groups of DOX against HeLa tumors in mice.

Sample	Tumor weight (g)	Inhibition rate (%)	Liver index	Spleen index
Normal saline	9.32 ± 0.41	−^a^	0.050 ± 0.006	0.013 ± 0.002
DOX	4.33 ± 0.26	53.59%	0.039 ± 0.004	0.004 ± 0.001
DOX-NPs	3.82 ± 0.38	59.01%	0.046 ± 0.005	0.012 ± 0.005
DOX-PDA-NPs	3.76 ± 0.47	59.66%	0.050 ± 0.008	0.014 ± 0.001
DOX-PDA-FA-NPs	2.72 ± 0.30	70.82%	0.051 ± 0.009	0.013 ± 0.004
DOX-PDA-RGD-NPs	2.32 ± 0.42	75.11%	0.051 ± 0.006	0.015 ± 0.003

^a^The data is not meaningful.

Systemic toxicity is a key factor to consider during cancer therapy and body weight is the common indicators of systemic toxicity (Hong et al., 2016). The changes of body weight during the treatment are shown in Figure 4(B(b)), the body weight of the saline control group was increased rapidly while PDA-coated nanoparticles raised gradually, which were due to the tumor quickly growth in saline control group. On the contrary, the body for the free DOX group, decreased significantly after the third injection while the tumor steadily grows (4.325 g). As for DOX-NPs group, the weight was almost unchanged. The results reflect that the relatively good safety of DOX nanoparticles, and proved the PDA film were nontoxicity, biocompatible and can reduce the toxicity of nanoparticles compared with DOX-NPs (Cheng et al., 2017a). Moreover, four mice of free DOX group died during the intravenous injection, and the other groups of mice all survived, which could further certify the strong toxicity of the free DOX group (Loomis et al., 2011). Taken together, all the above findings suggested that DOX-PDA-FA-NPs and DOX-PDA-RGD-NPs might be a good dosage form for DOX to treat cancer with low toxicity.

## Conclusions

In this study, novel PDA modified pH-sensitive DOX nanoparticles, with FA and RGD as targeting ligands, were developed for precise cervical cancer therapy. The DOX-PDA-FA-NPs and DOX-PDA-RGD-NPs showed small particle size, outstanding stability in various physiological media, high accumulation in tumor tissues, and pH sensitivity character. Targeting nanoparticles also achieved higher TIR than other nanoparticles; meanwhile, it decreased the side effects compared with free DOX. This study provides dopamine polymerization as a simple and versatile biocompatible platform for the design of drug delivery systems and could be used as the potential targeting carriers for cancer treatments in the future.

## Supplementary Material

IDRD_Han_Supplemental_Content.docx
