# Supplementary material for: Surface modification of doxorubicin-loaded nanoparticles based on polydopamine with pH-sensitive property for tumor targeting therapy
Source: Drug Deliv. 2018 Feb 19;25(1):564–75. doi: 10.1080/10717544.2018.1440447 (PMC6058689; doi:10.1080/10717544.2018.1440447)
Supplement: IDRD_Han_Supplemental_Content.docx [file IDRD_A_1440447_SM8577.docx]

Supplemental File

**Surface modification of doxorubicin-loaded nanoparticles based on polydopamine with pH-sensitive property for tumor targeting therapy**

Dongdong Bi^a^, Lei Zhao ^b^, Runqi Yu ^c^, Haowen Li ^a^, Yifei Guo ^a^, Xiangtao Wang ^a^* and Meihua Han ^a^*

*^a^**Institute of Medicinal Plant Development,* *Chinese Academy of Medical Sciences & Peking Union Medical College, Beijing, China; ^b^Life Science and Environmental Science Center, Harbin University of Commerce, Harbin, China; ^c^School of Pharmacy,* *Heilongjiang University of Chinese Medicine, Harbin, China*


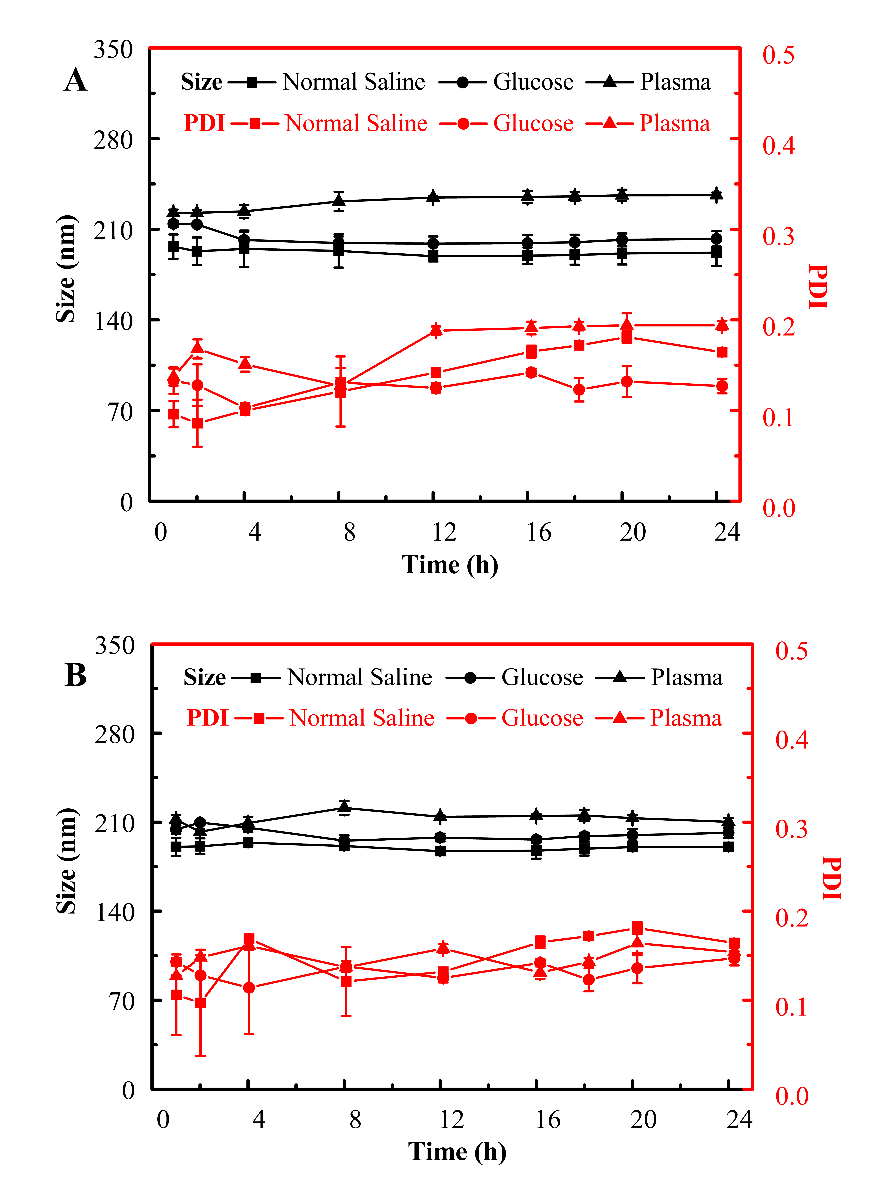


**Figure S1.** Stability of DOX-PDA-FA-NPs (A) and DOX-PDA-RGD-NPs (B) in physiological media (n=3).


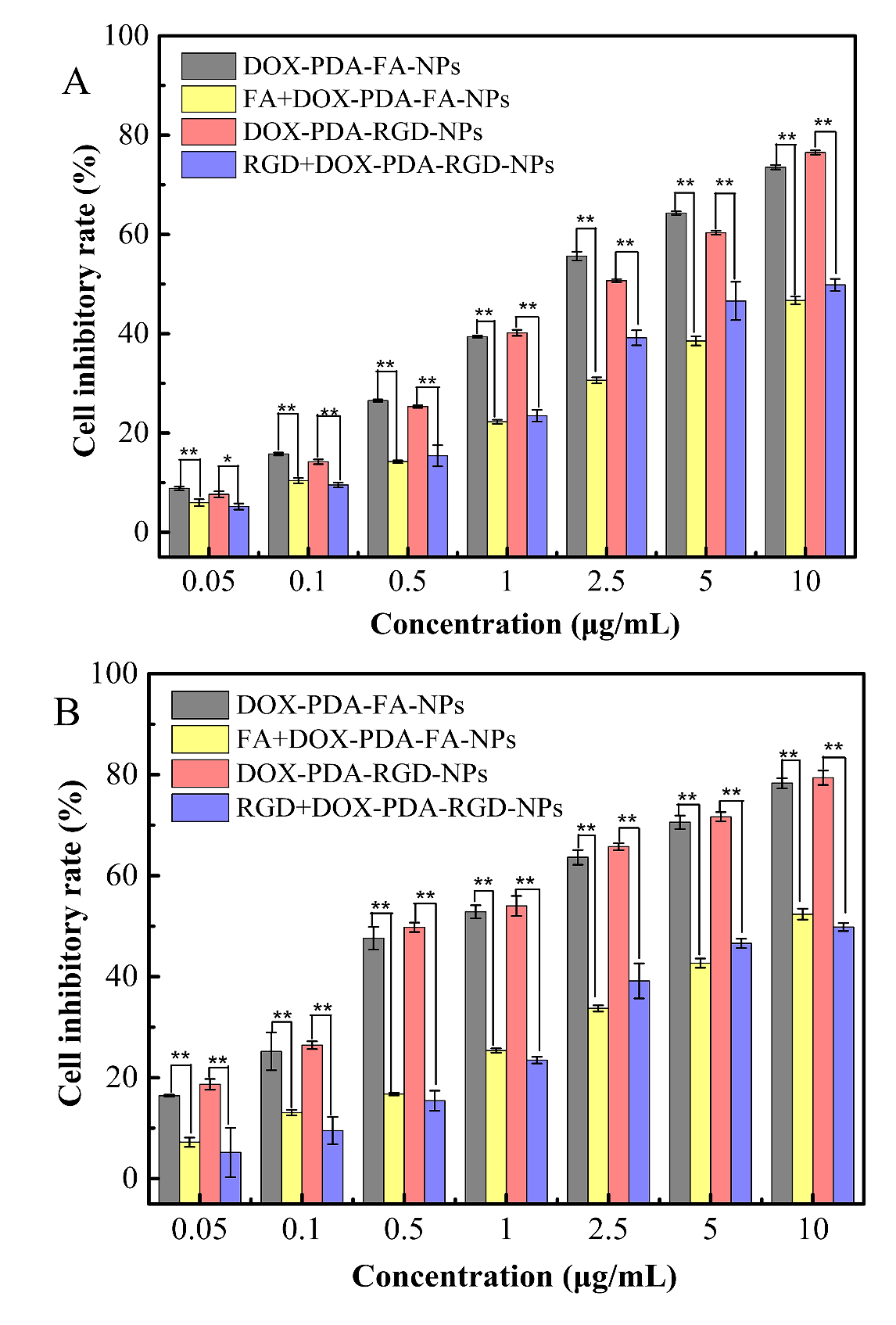


**Figure S2.** A: Cytotoxicity of free FA and RGD toward HeLa cells for 24 hours. B: Cytotoxicity of free FA and RGD toward HeLa cells for 48 hours (n=6), *p<0.05, **p<0.01.

**Table S1.** Characterization of DOX-NPs, DOX-PDA-NPs, DOX-PDA-RGD-NPs, and DOX-PDA-FA-NPs (n=3).

| **Samples (n=3)** | **Size (nm)** | **PDI** | **ZP (mV)** | **LC (%)** |
| --- | --- | --- | --- | --- |
| DOX-NPs | 162.01±0.90 | 0.19±0.00 | -6.96±0.45 | 4.98±0.26 |
| DOX-PDA-NPs | 185.62±2.56 | 0.18±0.01 | -14.74±2.34 | 4.87±0.21 |
| DOX-PDA-RGD-NPs | 194.97±1.33 | 0.11±0.04 | -15.4±1.57 | 4.46±0.15 |
| DOX-PDA-FA-NPs | 190.91±0.19 | 0.15±0.01 | -19.3±0.59 | 4.49±0.19 |

**Note:** Data presented as mean ± SD.
